# The epidemiology of road traffic injuries in the republic of Serbia: a study based on hospital data, 2015-2019

**DOI:** 10.3389/fpubh.2024.1468505

**Published:** 2024-11-15

**Authors:** Smiljana Rajčević, Mirjana Štrbac, Dragoslav Kukić, Marija Marković, Ivan Ivanović, Radmila Petrović, Ivana Radić

**Affiliations:** ^1^Institute of Public Health of Vojvodina, Novi Sad, Serbia; ^2^University of Novi Sad, Faculty of Medicine, Novi Sad, Serbia; ^3^Department of Traffic Engineering, Faculty of Economics and Engineering, Novi Sad, Serbia; ^4^City Institute of Pubic Health of Belgrade, Belgrade, Serbia; ^5^Institute of Public Health of Serbia “Dr Milan Jovanovic Batut”, Belgrade, Serbia; ^6^University Clinical Center of Vojvodina, Novi Sad, Serbia

**Keywords:** road traffic injuries, road traffic crash, hospitalization, hospital report, Serbia

## Abstract

**Introduction:**

Road traffic injuries (RTI) are the leading cause of death and severe disability among individuals under the age of 40, posing a significant public health challenge globally. This manuscript highlights key aspects of the epidemiology of injuries in road traffic crashes (RTC) in Serbia, based on hospitalization report data.

**Objectives:**

The main aim of this study was to analyze the epidemiological characteristics of road traffic injuries (RTI) based on hospital data over a five-year period in Serbia.

**Methods:**

The data for this study were obtained from the Hospitalization Report, which is part of the hospitalization database maintained by the Institute for Public Health of Serbia “Dr Milan Jovanović Batut,” covering the period from January 2015 to December 2019. The research included data from the Hospitalization Reports of 66 healthcare institutions across Serbia.

**Results:**

During the study period, a total of 15,028 patients with road traffic injuries were admitted to healthcare institutions in Serbia. During the five-year period, the crude RTI incidence rate increased every year, from 39.0/100, 000 in 2015 to 43.7/100,000 in 2019. Older adult people aged 65 and over were particularly vulnerable as bicyclists and pedestrians (31.3, 27.7%, respectively). The Vojvodina region experienced a higher incidence of injuries among bicyclists compared and car accidents were most frequent in Central Serbia than in the other regions of Serbia. Craniocerebral injuries were the most common type of road traffic injury, accounting for 37.8% of cases. Significant differences in the types of injuries were observed based on age (*χ*^2^ = 649.859; *p* < 0.001) and gender (*χ*^2^ = 31.442; *p* < 0.001).

**Conclusion:**

Understanding the epidemiological profile of road users involved in accidents is essential for monitoring and controlling specific risk factors. Our results highlight the need for enhanced traffic safety measures at the local level.

## Introduction

RTI are a leading cause of death and disability worldwide, particularly in low-and middle-income countries. According to WHO, over 1.35 million people die in traffic accidents each year, equating to one death every 25 s. These accidents impose a global financial burden measured in billions of dollars (1, 2). In addition to the physical injuries, road traffic accidents also have profound psychological consequences for the victims and their families. A significant proportion of accident victims develop psychological disorders after a road accident traffic accidents such as post-traumatic stress disorder (PTSD), major depressive disorder, fear of driving, and other anxiety disorders (3, 4). Research shows that victims do not recover to their pre-accident condition for several years after a traffic accident (5, 6).

According to data from the Traffic Safety Agency of Serbia, between 2018 and 2022, a total of 2,648 people were killed in road traffic accidents (RTA) in the Republic of Serbia (RS), with 16,251 sustaining serious injuries and 81,158 experiencing minor injuries. During the period from 2010 to 2019, Serbia recorded a long-term decline in road traffic fatalities, decreasing from 13.9 deaths per 100,000 inhabitants in 2010 to 7.8 per 100,000 in 2019, representing a total reduction of 48%. Despite this progress, Serbia remains significantly above the EU average of 46 road traffic fatalities per million inhabitants in 2022 (7–9). Furthermore, road deaths increased in 19 PIN countries between 2021 and 2022, in Serbia increased from 75 to 83 road deaths per million inhabitants respectively, for the same period (9, 10). Serbia’s traffic environment presents several notable characteristics, particularly concerning infrastructure, the vehicle fleet, and mobility patterns. While Serbia has a well-developed road network, many local and regional roads remain in poor condition. The highway network is expanding, with recent investments focused on connecting major cities such as Belgrade, Novi Sad, and Niš, as well as key international borders. However, the infrastructure for cycling and pedestrian traffic, while improving, still lags behind European standards, with ongoing efforts to expand bike lanes and pedestrian-friendly zones. The average age of vehicles in Serbia is relatively high, at approximately 15 years, largely due to the prevalence of imported used cars, which are more affordable. Research has shown that older vehicles and poorly maintained roads are associated with higher accident severity and mortality rates. Serbia currently has over 2.5 million registered vehicles, the majority of which are passenger cars. Urbanization and economic growth have contributed to a steady rise in vehicle numbers, further straining the existing infrastructure. In larger cities like Belgrade, Novi Sad, and Niš, public transportation is primarily bus-based, with Belgrade also operating trams. However, the vehicle fleets are aging and in need of modernization. Increasing vehicle numbers, especially in urban areas, combined with insufficient infrastructure, have exacerbated congestion, raising the risk of accidents, particularly in cities like Belgrade where traffic jams are frequent during peak hours. Intercity bus services form the backbone of public transport, connecting cities and towns across Serbia, with additional routes to neighboring countries. In contrast, Serbia’s rail network is less developed, hampered by an aging fleet and underinvestment in infrastructure (11, 12).

The high rate of traffic-related injuries and fatalities in Serbia is recognized by the European Union (EU) as a significant public health concern. In response, the EU initiated a comprehensive five-year project (2019–2023) aimed at improving road safety in the country. By implementing targeted interventions and aligning road safety measures with EU standards, the project seeked to reduce traffic-related injuries and fatalities, promote safer driving behaviors, and foster a culture of safety among all road users in Serbia. This study was conducted as part of the EU-funded project “Improving Road Safety in Serbia (13). This is the first time that this problem is being approached in a multisectoral manner in our country, recognizing that RTA are not only a problem for the police and traffic experts but also require the involvement of healthcare in injury prevention.

In the past 10 years, the Republic of Serbia has significantly improved legal regulations related to traffic safety through the adoption of important legal and strategic documents, such as the Law on Road Traffic Safety. To improve traffic safety in the RS and achieve the goal of reducing the number of traffic accidents by 50%, the Government adopted the Traffic Safety Strategy of the Republic of Serbia for the period from 2023 to 2030, along with an action plan for 2023 to 2025 (14).

Studies around the world have shown that no single database on RTI, if used independently, without insight into other databases, provides enough information to obtain a complete picture of serious traffic injuries and to fully understand all injury mechanisms (15–17). Police databases are mainly based on the technical aspects of the traffic accident and do not contain a precise definition of the injury, and hospital databases that precisely define the type, degree, or severity of the injury do not contain details about the manner in which the traffic accident occurred. World experts agree that the best way to obtain quality data is to combine and combine data from different databases. Sweden or the Netherlands where combining police and healthcare data has led to significant improvements in road safety analysis (18, 19). Thus, the use of data from the health sector to classify and qualify injuries is necessary to complement police data and obtain an optimal data set for defining a serious injury and vice versa (8, 15, 16, 20).

### Specific objectives and hypotheses of the study

The main aim of this study was to analyze the epidemiological characteristics of RTI based on hospital data over a five-year period in Serbia. The specific objectives were: (1) To analyze the incidence of RTIs across different regions of the Republic of Serbia from 2015 to 2019 using hospital data. (2) To examine the demographic characteristics of patients hospitalized due to RTIs and identify high-risk groups frequently affected by road traffic accidents (RTAs). (3) To analyze the external causes of injuries, categorized by gender and age. (4) To determine the most common types of injuries based on anatomical location, gender, and age.

### Main research hypotheses

(1) There are regional differences in the incidence of road traffic injuries RTI across Serbia. (2) The frequency of RTI varies across age groups, with vulnerable road users including the older adult, children, and young drivers. (3) The proportion of male patients hospitalized after RTA is significantly higher than that of female patient. (4) The most common injuries among hospitalized traffic accident patients are head and limb injuries. (5) Car occupants constitute the majority of hospitalized patients when categorized by the external cause of injury.

These hypotheses and specific objectives provide a foundation for conducting a comprehensive epidemiological analysis of RTIs in Serbia.

## Methods

This study is a retrospective analysis of data for patients hospitalized due to RTI in 66 hospitals in the Republic of Serbia from January 2015 to December 2019.

The data was obtained from the Hospitalization report, sourced from the hospitalization database of the Institute for Public Health of Serbia “Dr Milan Jovanović Butut,” initiated by the Ministry of Health.

The Hospitalization report is an individual statistical report, filled in when a patient is admitted for treatment in an inpatient health facility. The dataset obtained from these reports provides the necessary elements for relevant health-statistical analyses. Each hospitalization report is routinely filled out for every hospitalized patient with traffic accident injury information coded using the International Classification of Disease (ICD-10) with S or T codes from Chapter XIX (Injury, poisoning and certain consequences of external causes) for the type of injury and V01-V99 codes from Chapter XX (External causes of morbidity and mortality) for the external cause of injury. These reports are collected by district public health institutions and are primarily generated directly into the hospitalization database (World Health Organization).

For every person treated in hospitals Hospitalization Report is completed at the discharge of the patient. To determine how much hospitals are burdened with patients hospitalized after a traffic accident, the cumulative incidence was calculated, representing the number of hospitalized patients over a five-year period per 100,000 inhabitants.

In this study we collected data on the demographic characteristics of patient, type of injury, external cause of injury, an performed an analysis of injured patients according to districts. For the purpose of analysis, patients were divided into five groups based on the road-user category: pedestrian (V01-V09), bicyclist (V10-V19), motorcyclists (V20-V29), car occupants (V40-V49) and all other transport accidents (V30-V39, V50-V59, V60-V69, V70-V79, V80-V89, V90-V94, V95-V97, V98, V99). The car occupants and motorcyclists’ groups included both drivers and passengers.

The annual incidence rates of RTI were measured per 100,000 inhabitants. The numerator was the number of the reported injured cases, and the denominator was the total population of Serbia according to the 2011 census (21).

The statistical program SPSS (Statistical Package for Social Sciences), version 21.0, was used for statistical analysis. Descriptive statistics methods were used to describe the sample characteristics. Absolute values and percentages were used for the presentation of categorical variables. The *χ*^2^ test was used to test the differences in the frequencies of the type of injury and the external causes of injury between different genders and age groups. Values of *p* < 0.05 were considered statistically significant.

Ethical considerations: Data used for this study are collected as part of a routine public health surveillance of hospital discharge reports. This surveillance is regulated by specific legislation of the Republic of Serbia (Law on health documentation and records in the field of health care) (22). All data are completely anonymized before being used for research purposes. To anonymize the data, direct identifiers were excluded from the database, and all unnecessary indirect data were also removed. All data are presented summarized and none of the data can be linked to specific individuals.

## Results

A total of 15,028 road traffic injury (RTI) patients were admitted to healthcare institutions in Serbia during the study period (2015–2019). The findings revealed that men were more prone to injuries, with a male-to-female ratio of 2:1 (*N* = 10,282, 68.4%; *N* = 4,746, 31.6%). The mean age of the entire sample was 44.3 years, with a standard deviation of 22.3 years (±22.3). The youngest patient was less than 1 year old, and the oldest patient was 97 years old.

Car accidents were the most frequent type of incident, accounting for nearly one-third (30.9%) of hospitalized patients. The age groups 30–49 (24%) and young road users aged 15–29 years (22.7%) represented the majority of cases. The demographic and injury characteristics of the study sample are detailed in Table 1.

**Table 1:** Demographic and injury characteristics of the study sample, Serbia, period 2015–2019.

	Number (*N*)	Percent (%)
Mean age (years), SD	41.43 (+/−22.3)	
Sex		
Men	10,282	68.4
Women	4,746	31.6
Age (years)		
0–6	553	3.7
7–14	1,451	9.7
15–19	1,229	8.2
20–29	2,177	14.5
30–39	1,812	12.1
40–49	1,774	11.8
50–64	3,267	21.7
65+	2,765	18.4
Type of injury		
Head and neck injuries (S00-S19)	5,687	37.8
Injuries to the thorax (S20-S29)	2,266	15.1
Upper extremities injuries (S40-S69)	1,481	9.9
Injuries to the abdomen, lower back, lumbar spine, pelvis and external genitals (S30-S39)	1,693	11.3
Lower extremities injuries (S70-S99)	3,553	23.6
Other (T00-T98)	348	2.3
External cause of injury		
Pedestrian injured in transport accident (V01-V09)	2,658	17.7
Cyclist or motorcyclist injured in transport accident (V10-V29)	3,723	24.8
Motorcycle rider injured in transport accident (V20-V29)	1,806	12
Car occupant injured in transport accident (V40-V49)	4,639	30.9
All other transport accidents (V30-V39, V50-V59, V60-V69, V70-V79, V80-V89, V90-V94, V95-V97, V98-V99)	2,202	14.7
Number of RTI hospitalized patients by year		
2015	2,803	18.7
2016	2,847	18.9
2017	3,090	20.6
2018	3,142	20.9
2019	3,146	20.9
Total	**15,028**	**100.0**

Bold are total number of participants and names of variables.

**Figure 1:**
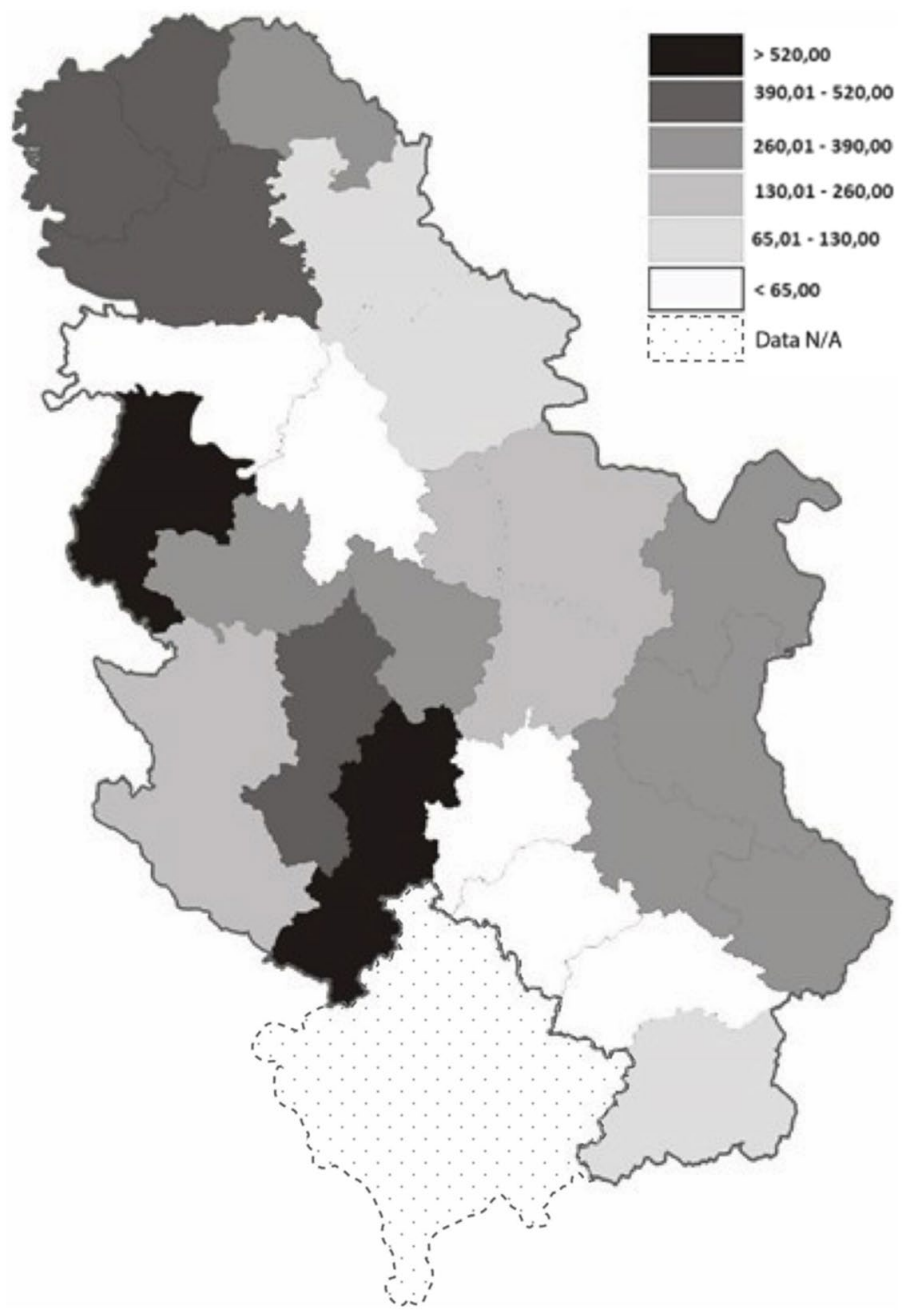
Road traffic injury incidence of hospitalized patients in Administrative districts in Serbia, period 2015–2019.

During the 5-year period, the crude RTI incidence rate increased every year, from 39.0/100, 000 in 2015 to 43.7/100,000 in 2019. The five-year mean RTI incidence was 41.8/100,000. The highest RTI incidence rates of hospitalized persons, with values over 500 per 100,000 inhabitants, were registered in three districts of Serbia (Raski, Macvanski, and West Backa districts). They were followed by the Moravian, South Backa, and North Backa districts that had incidence rates ranging from 400 to 500 per 100,000 inhabitants (Figure 1).

According to hospital data car occupants accounted the majority of hospitalized patients. Of the total number hospitalized due to RTI, 4,639 (30.9%) were car occupants, 3,723 (24.8%) were bicyclist, 2,658 (17.7%) were pedestrian and 1,806 (12%) were motorcyclists. Vulnerable road users (pedestrians, cyclists and motorcyclist) accounted half 8,187 (54.5%) of the injuries over the 5-year period. The results showed that car occupants, bicyclist as well as pedestrians, were the most affected road users in all three regions (30.9, 24.8, 17.7%). However, bicyclists were more affected in Vojvodina region than in the other two regions. Hospital data showed that every third patient (33.1%) in the Vojvodina region was injured as bicyclist at RTC, compared to Belgrade region and Central Serbia where patients were mostly affected as car passengers (25.8 and 35.1%, respectively). Generally, motorcyclists were the least affected road user (Table 2).

**Table 2:** Distribution of external causes of injury according to region, Serbia, period 2015–2019.

Region	External cause of injury												
	Pedestrian injured in transport accident (V01-V09)		Pedal cyclist injured in transport accident (V10-V19)		Motorcycle rider injured in transport accident (V20-V29)		Car occupant injured in transport accident (V40-V49)		All other transport accidents		Total		p*
	*N*	%	*N*	%	*N*	%	*N*	%	*N*	%	*N*	%	
Vojvodina	947	17.9	1,755	33.1	726	13.7	1,291	24.4	578	10.9	5,297	100.0	<0.001
Belgrade Region	182	25.1	105	14.5	132	18.2	187	25.8	118	16.3	724	100.0	
Central Serbia	1,529	17	1,863	20.7	948	10.5	3,161	35.1	1,506	16.7	9,007	100.0	
Total	2,658	17.7	3,723	24.8	1,806	12.0	4,639	30.9	2,202	14.7	15,028	100.0	

*χ^2^ test.

There were significant differences in types of injuries according to external cause of injury (*χ*^2^ = 1,208,054; *p* < 0.001). The most common injury among pedestrians injured in transport accidents and among motocyclists was lower extremities injuries. Among car occupants and cyclist, the most frequent injuries were head and neck injuries (Table 3).

**Table 3:** Type of injury according to external cause of injury, hospitalized patients, Serbia, period 2015–2019.

External cause of injury	Type of injury														
	Head and neck injuries (S00-S19)		Injuries to the thorax (S20-S29)		Injuries to the abdomen, lower back, lumbar spine, pelvis and external genitals (S30-S39)		Upper extremities injuries (S40-S69)		Lower extremities injuries (S70-S99)		Other injuries (T00-T98)		Total		p*
	*N*	%	*N*	%	*N*	%	*N*	%	*N*	%	*N*	%	*N*	%	
Pedestrian injured in transport accident (V01-V09)	828	31.2	195	7.3	300	11.3	249	9.4	1,014	38.1	72	2.7	2,658	100.0	<0.001
Pedal cyclist injured in transport accident (V10-V19)	1,563	42.0	304	8.2	362	9.7	452	12.1	974	26.2	68	1.8	3,723	100.0	
Motorcycle rider injured in transport accident (V20-V29)	557	30.8	244	13.5	184	10.2	227	12.6	549	30.4	45	2.5	1,806	100.0	
Car occupant injured in transport accident (V40-V49)	2,012	43.4	972	21.0	556	12.0	384	8.3	602	13.0	113	2.4	4,639	100.0	
All other transport accidents	727	33.0	551	25.0	291	13.2	169	7.7	414	18.8	50	2.3	2,202	100.0	
Total	5,687	37.8	2,266	15.1	1,693	11.3	1,481	9.9	3,553	23.6	348	2.3	15,028	100.0	

Epidemiological characteristics of road users by age showed that children aged 7–14 were more affected as pedestrian or bicyclist compared to children younger than 7 years who were more often injured as car passenger and bicyclist. Among the age group 15–64 years old, car occupants were the most affected by RTC. Older adult people above 65 were particularly vulnerable as bicyclist and pedestrians (31.3, 27.7%, respectively) in RTC (Table 4).

**Table 4:** Distribution of external causes of injury according to age and gender of the hospitalized patient, Serbia, period 2015–2019.

Gender	Age	External cause of injury												
		Pedestrian injured in transport accident (V01-V09)		Pedal cyclist injured in transport accident (V10-V19)		Motorcycle rider injured in transport accident (V20-V29)		Car occupant injured in transport accident (V40-V49)		All other transport accidents		Total		p*
		*N*	%	*N*	%	*N*	%	*N*	%	*N*	%	*N*	%	
Total														
	0–6	144	26.0	186	33.6	8	1.4	169	30.6	46	8.3	553	100.0	<0.001
	7–14	390	26.9	767	52.9	50	3.4	179	12.3	65	4.5	1,451	100.0	
	15–19	232	18.9	234	19.0	194	15.8	431	35.1	138	11.2	1,229	100.0	
	20–29	211	9.7	266	12.2	400	18.4	985	45.2	315	14.5	2,177	100.0	
	30–39	167	9.2	266	14.7	348	19.2	730	40.3	301	16.6	1,812	100.0	
	40–49	219	12.3	302	17.0	274	15.4	652	36.8	327	18.4	1,774	100.0	
	50–64	529	16.2	836	25.6	363	11.1	954	29.2	585	17.9	3,267	100.0	
	65+	766	27.7	866	31.3	169	6.1	539	19.5	425	15.4	2,765	100.0	
	Total	2,658	17.7	3,723	24.8	1,806	12.0	4,639	30.9	2,202	14.7	15,028	100.0	
Male														
	0–6	94	27.2	132	38.3	5	1.4	89	25.8	25	7.2	345	100.0	<0.001
	7–14	219	22.2	579	58.8	42	4.3	106	10.8	39	4.0	985	100.0	
	15–19	108	13.7	172	21.9	156	19.8	261	33.2	90	11.4	787	100.0	
	20–29	138	8.1	192	11.3	364	21.4	748	44.1	255	15.0	1,697	100.0	
	30–39	109	8.0	192	14.1	319	23.4	498	36.3	242	17.8	1,360	100.0	
	40–49	128	10.3	199	16.1	250	20.2	422	34.1	239	19.3	1,238	100.0	
	50–64	245	11.5	534	25.1	299	14.1	589	27.7	458	21.6	2,125	100.0	
	65+	345	19.8	604	34.6	149	8.5	328	18.8	319	18.3	1,745	100.0	
	Total	1,386	13.5	2,604	25.3	1,584	15.4	3,041	29.6	1,667	16.2	10,282	100.0	
Female														
	0–6	50	24.0	54	26.0	3	1.4	80	38.5	21	10.1	208	100.0	<0.001
	7–14	171	36.7	188	40.3	8	1.7	73	15.7	26	5.6	466	100.0	
	15–19	124	28.1	62	14.0	38	8.6	170	38.5	48	10.9	442	100.0	
	20–29	73	15.2	74	15.4	36	7.5	237	49.4	60	12.5	480	100.0	
	30–39	58	12.8	74	16.4	29	6.4	232	51.3	59	13.1	452	100.0	
	40–49	91	17.0	103	19.2	24	4.5	230	42.9	88	16.4	536	100.0	
	50–64	284	24.9	302	26.4	64	5.6	365	32.0	127	11.1	1,142	100.0	
	65+	421	41.3	262	25.7	20	2.0	211	20.7	106	10.4	1,020	100.0	
	Total	1,272	26.8	1,119	23.6	222	4.7	1,598	33.7	535	11.3	4,746	100.0	

*χ^2^ test.

Regarding the spectrum of injuries among the study population, craniocerebral trauma was the most frequent RTI (37.8%), followed by upper and lower extremities injuries (33.5% in total) and thoracic injury (15.1%). The analysis of the structure of the type of injuries among different age categories showed that head injuries had a significantly higher participation in younger age categories than in older ones. Lower extremities injuries were more prevalent among older patients than in younger. Among children aged 0–6 every seventh injury was lower extremity injury (14.1%), while among persons aged 65 and over every third injury was lower extremities injury (33.6%) (Table 5).

**Table 5:** Type of injury in hospitalized patients according after road traffic injuries, Serbia, period 2015–2019.

Gender	Age	Type of injury														
		Head and neck injuries (S00-S19)		Injuries to the thorax (S20-S29)		Injuries to the abdomen, lower back, lumbar spine, pelvis and external genitals (S30-S39)		Upper extremities injuries (S40-S69)		Lower extremities injuries (S70-S99)		Other injuries (T00-T98)		Total		p*
		*N*	%	*N*	%	*N*	%	*N*	%	*N*	%	*N*	%	*N*	%	
Total																
	0–6	338	61.1	18	3.3	55	9.9	47	8.5	78	14.1	17	3.1	553	100.0	<0.001
	7–14	750	51.7	43	3.0	197	13.6	205	14.1	221	15.2	35	2.4	1,451	100.0	
	15–19	620	50.4	89	7.2	150	12.2	135	11.0	201	16.4	34	2.8	1,229	100.0	
	20–29	978	44.9	278	12.8	229	10.5	205	9.4	439	20.2	48	2.2	2,177	100.0	
	30–39	646	35.7	301	16.6	224	12.4	189	10.4	412	22.7	40	2.2	1,812	100.0	
	40–49	601	33.9	354	20.0	206	11.6	181	10.2	388	21.9	44	2.5	1,774	100.0	
	50–64	926	28.3	707	21.6	371	11.4	307	9.4	885	27.1	71	2.2	3,267	100.0	
	65+	828	29.9	476	17.2	261	9.4	212	7.7	929	33.6	59	2.1	2,765	100.0	
	Total	5,687	37.8	2,266	15.1	1,693	11.3	1,481	9.9	3,553	23.6	348	2.3	15,028	100.0	
Male																
	0–6	208	60.3	15	4.3	37	10.7	33	9.6	42	12.2	10	2.9	345	100.0	<0.001
	7–14	498	50.6	30	3.0	129	13.1	150	15.2	151	15.3	27	2.7	985	100.0	
	15–19	391	49.7	56	7.1	90	11.4	96	12.2	135	17.2	19	2.4	787	100.0	
	20–29	743	43.8	235	13.8	163	9.6	166	9.8	348	20.5	42	2.5	1,697	100.0	
	30–39	465	34.2	235	17.3	140	10.3	151	11.1	337	24.8	32	2.4	1,360	100.0	
	40–49	412	33.3	266	21.5	144	11.6	127	10.3	258	20.8	31	2.5	1,238	100.0	
	50–64	637	30.0	496	23.3	244	11.5	181	8.5	514	24.2	53	2.5	2,125	100.0	
	65+	564	32.2	361	20.7	163	9.3	98	5.6	519	29.7	40	2.3	1,745	100.0	
	Total	3,918	38.1	1,694	16.5	1,110	10.8	1,002	9.7	2,304	22.4	254	2.5	10,282	100.0	
Female																
	0–6	130	62.5	3	1.4	18	8.7	14	6.7	36	17.3	7	3.4	208	100.0	<0.001
	7–14	252	54.1	13	2.8	68	14.6	55	11.8	70	15.0	8	1.7	466	100.0	
	15–19	229	51.8	33	7.5	60	13.6	39	8.8	66	14.9	15	3.4	442	100.0	
	20–29	235	49.0	43	9.0	66	13.8	39	8.1	91	19.0	6	1.3	480	100.0	
	30–39	181	40.0	66	14.6	84	18.6	38	8.4	75	16.6	8	1.8	452	100.0	
	40–49	189	35.3	88	16.4	62	11.6	54	10.1	130	24.3	13	2.4	536	100.0	
	50–64	289	25.3	211	18.5	127	11.1	126	11.0	371	32.5	18	1.6	1,142	100.0	
	65+	264	25.9	115	11.3	98	9.6	114	11.2	410	40.2	19	1.9	1,020	100.0	
	Total	1,769	37.3	572	12.1	583	12.3	479	10.1	1,249	26.3	94	2.0	4,746	100.0	

*χ^2^ test.

**Figure 2:**
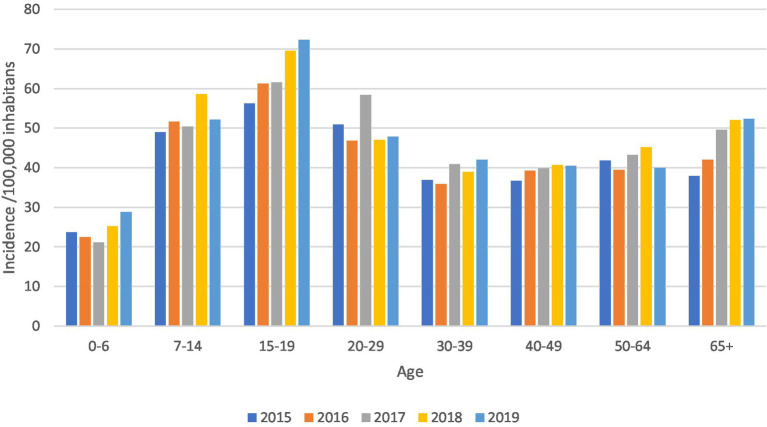
Age-specific incidence of RTI in hospitalized patients/100,000 inhabitants, Serbia, period 2015–2019.

According to the obtained data, the highest age specific incidence of RTI was registered among young people aged 15–19 (Figure 2).

## Discussion

RTIs remained a significant public health issue, reflecting both global and regional trends in road safety. Understanding the epidemiology of injuries in RTC in Serbia is crucial for developing evidence-based interventions that can effectively reduce the burden of RTI. To our knowledge, this is the first study that analyzed the results on hospital data on RTI in Serbia. This manuscript highlights key aspects of the epidemiology of injuries in RTC in Serbia, based on hospitalization report data.

Serbia has experienced a concerning number of RTA with 170,090 recorded between 2018 and 2022, contributing to a high incidence of injuries. RTI remain a significant public health concern globally, and Serbia is no exception, with a five-year mean RTI incidence rate of 41.82 per 100,000 population. Our study revealed substantial disparities in RTI incidence across different regions of Serbia, confirming the hypothesis that regional differences exist and that the incidence fluctuated during the observed period without a significant reduction. The districts of Raški, Mačvanski, and West Bačka recorded the highest cumulative incidence rates based on hospitalized data. These geographical variations are likely influenced by factors such as road conditions, socioeconomic indicators, and geographical location, as these districts are border areas with frequent traffic and poor road infrastructure. In terms of road safety, Serbia’s roads are considered among the least safe in Europe. According to the World Economic Forum’s Global Competitiveness Report from 2019, Serbia ranked 33rd in road quality, with a score of 3.5. Countries with lower scores include Moldova (2.6), Bosnia and Herzegovina (2.8), Romania and Ukraine (3.0 each), and Bulgaria and North Macedonia (3.4 each) (23). During the study period (2015–2019), a total of 15,028 patients were admitted to healthcare institutions in Serbia for treatment following RTA. The number of RTI requiring hospitalization represents a substantial economic burden in Serbia, an upper-middle-income country (24). The total economic costs of road crashes in Serbia in 2017 amounted to EUR 272.1 million, or 0.7% of Serbia’s gross domestic product (GDP) (8). A study of RTI in Poland found that road traffic crashes equal 2.1% of (GDP) (25). Wachnicka et al. confirmed the impact of regional factors on road safety, identifying several statistically significant variables. These include gross domestic product *per capita* (GDPPC), the number of passenger cars per inhabitant (MRPC), the share of passenger vehicles (PPC), and life expectancy at birth (LIFE). Additionally, factors related to regional borders, such as inner-border (IB) and outer-border (OB) areas, were also found to be influential (26). Other researchers have also affirmed that road safety varies significantly between regions (27–30). Additionally, a study by Li et al. identified improper vehicle operation, speeding, loss of vehicle control, and inefficient driver management as the top four risk factors in the comprehensive evaluation of road safety risk. These findings align with other studies confirming the regional disparities in road safety outcomes (31).

The data indicate that the average age of hospitalized patients due to RTI in our sample was 41.4 years. This is higher than the average age reported in similar studies from Greece, Poland, Bangladesh, and Iran, where the average ages were 35.4, 36.2, 36.7, and 33.8 years, respectively (25, 32–34). In Serbia, the most frequently hospitalized individuals following road traffic collisions (RTCs) were those aged 50–64 (21.7%), followed by individuals aged 65 and older (18.4%), together comprising 40.1% of the total. The demographic aging of Serbia’s population is reflected in the lower proportion of younger people and the increasing share of older adult individuals. With 22.3% of the population aged 65 and older and an average age of 43.9, Serbia’s population is continually aging. This trend suggests that the burden of RTA on the healthcare system is likely to increase in the future (12). Similar findings, showing an increasing trend in the hospitalization of older adult drivers, were observed in studies by Goldman et al. and other researchers (35, 36).

The public health significance of RTA in Serbia is highlighted by the impact on children and young people, one of the most vulnerable groups. Data show that 36.1% of hospitalized RTA patients are children aged 0–14 and young people aged 15–29. The age distribution of those injured, along with findings from a study on non-fatal RTAs in Riyadh (37), emphasizes the urgent need for prevention programs. These programs should prioritize early awareness and education initiatives targeting both young drivers and passengers of all genders to improve road safety and reduce accident rates. A study conducted in India on the patterns of RTI and pre-hospitalization factors found that males and young adults aged 18–34 were the most affected in both urban and rural settings. In urban areas, most accident victims did not require hospitalization. However, in rural areas, a significant number of accidents occurred on national highways or rural roads, with most injuries resulting from routine, straight driving. Notably, 43.9% of these rural cases required hospitalization, indicating a higher injury severity in these areas (37–39). Conversely, a study in Isfahan Province found a higher number of fatal crashes in the provincial capital compared to other cities (40).

A review of the literature indicates that significantly more men than women are injured in RTA, with the exception of pedestrian groups aged 15–19 and over 65, where women constitute the majority. Similar studies have confirmed a higher incidence of injuries among women as pedestrians in RTA (34, 41). In our study, the male-to-female ratio was 2:1, confirming our hypothesis regarding gender differences among hospitalized patients following RTA. The data indicate that males are disproportionately represented among hospitalized RTI patients, which may be attributed to a higher likelihood of engaging in risky driving behaviors. Numerous studies have consistently shown that men face a greater risk of involvement in RTA (1, 42, 43).

Car occupants represented the majority of hospitalized patients, accounting for 30.9% of cases, while approximately one-fourth (24.8%) of hospitalized patients were injured in cycling accidents. Significant differences were observed in the age distribution concerning external causes of injuries. Children aged 0–14 and individuals aged 65 and older were primarily injured as pedal cyclists. In contrast, the most common external causes of injuries among the age categories 15–19, 20–29, 30–39, 40–49, and 50–64 were car accidents. Previous studies have demonstrated that vulnerable age groups, such as children and the older adult, are more susceptible to severe injuries, highlighting them as key focus groups for prevention efforts (44–46). Based on our study results, we confirmed the hypothesis that children, young drivers, and the older adult constitute a vulnerable category of road users. Similar findings were reported in studies conducted in Romania, which is the only country included in the Road Safety PIN report from 2012 that documented an increase in the number of young people killed on the roads (47). Young adults required emergency care for injuries, including road traffic injuries (RTIs), more frequently than for any other health condition (48, 49). Our data indicate that individuals aged 18 to 29 account for the highest proportion of emergency department visits due to RTIs, comprising 30.5% (*n* = 219) of cases. These findings are consistent with previous studies that support this trend (25, 50).

Certain demographic factors significantly influence the epidemiology of RTI in Serbia. Young adults, particularly males, are prone to engaging in risky behaviors, such as excessive speed, driving under the influence of alcohol or illicit drugs, and distractions from mobile phone use while driving. These behaviors are notably more prevalent in this age group, highlighting the urgent need for targeted interventions (8, 51). Multiple studies support the effectiveness of communication campaigns as a complement to road safety education and law enforcement, serving as a key element in reducing the severity of crash consequences. Such initiatives could have a positive impact on road safety prevention in the country (52–54). Additionally, Klinjun et al. identify several preventable risk factors associated with traffic injuries (55). Furthermore, bicyclists are particularly affected in the Vojvodina region, where bicycle usage is significantly higher than in other parts of Serbia. Understanding these demographic patterns can inform the development of public health campaigns and educational initiatives tailored to address specific high-risk groups (56). Our findings indicate that head and neck injuries (37.8%) and extremity injuries (33.5%) are the most prevalent among road traffic injuries (RTIs) in Serbia. The types of injuries observed in our study are consistent with those reported in another research (57, 58). Additionally, our data show that car occupants and cyclists face an increased risk of head and neck injuries compared to other groups of road users. The high percentage of head injuries among cyclists may be attributed to the low prevalence of protective equipment, such as helmets, in Serbia. Multiple studies have confirmed the effectiveness of helmet use in reducing mortality and injury severity following RTA (59–61). Baker et al. report that pedestrians and cyclists are at the greatest risk of severe injuries (62). In Europe, nearly 70% of all RTA fatalities involve a head injury, with 32% resulting from isolated head injuries (63). Furthermore, car occupants are at a higher risk of sustaining thoracic injuries, particularly in older age groups. The elevated frequency of thoracic injuries among car occupants may be attributed to the low percentage of seat belt usage, which aligns with findings from other reported studies (64, 65). In Benhamed et al.’s study, thoracic injuries were also identified as the most common type of injury among car occupants, accounting for 52.3% of cases (66). Lower extremity injuries were more prevalent among pedestrians, while cyclists and motorcyclists were more frequently exposed to upper extremity injuries. A previous study involving pedestrians and cyclists demonstrated that the lower extremities were the most commonly injured, followed by the upper extremities, which is consistent with our findings in this vulnerable road user group (67).

Understanding the epidemiological profile of road users involved in accidents is essential for monitoring and controlling specific risk factors. Analyzing hospital data on RTI enables the prioritization of preventive measures and the enhancement of emergency response protocols. These measures may include stricter law enforcement, public awareness campaigns promoting responsible driving behavior, improvements in road infrastructure, and the enhancement of emergency medical services. Through collaborative efforts between healthcare authorities, policymakers, and community stakeholders, targeted interventions can be implemented to reduce the incidence of RTIs and mitigate their adverse effects on public health and well-being. For future research on the epidemiological characteristics of traffic trauma in Serbia based on hospital data, we recommend the following key points:

- Data Collection and Standardization: Establish a national registry for injuries. Linking hospital data with emergency medical services, ambulance records, police reports, and insurance company databases can enhance the collection of accurate and comprehensive information.
- Analysis of Demographic Characteristics of the Injured: Investigate the demographic factors (such as gender, age, and socioeconomic status) associated with road traffic accidents (RTAs) in greater detail, focusing on variations among different groups, particularly vulnerable road users.
- Type and Severity of Injuries: Implement the Maximum Abbreviated Injury Scale (MAIS 3+) to assess the severity of injuries.
- Geographical Distribution of Traffic Accidents: Analyze the geographical distribution of traffic accidents and identify “black spots” (locations with a high risk of accidents) using hospital data.
- Research on Traffic Behavior of Participants: Conduct studies on the behaviors of road users, including driving speed, the use of safety systems, and compliance with regulations.
- Economic and Social Impact of Traffic Trauma: Investigate the economic costs associated with treating traffic injuries (both direct and indirect) as well as the long-term impact on families and communities.

This framework can serve as a base for the development of future research projects in Serbia, with the aim of improving traffic safety and reducing the rate of injuries and fatalities.

### Limitations

There are several limitations to our study. First, hospitalization reports RTI patients provide minimal or no information on the circumstances or additional risk factors contributing to the severity of road traffic accidents, such as accident causes, participant behavior, or weather and road conditions. Second, data on final outcomes are incomplete. There is a lack of information regarding the severity of injuries and the long-term consequences for each patient, such as disability, quality of life, or the enduring economic impact on families. Third, the study did not include there data on fatalities, including the characteristics of those who died and cases where deaths occurred outside the hospital setting or did not require hospitalization. Fourth, the absence of data on hospital length of stay (number of days) limits insights into the healthcare system’s burden resulting from traffic injuries. Recommendations for further research include analyzing the duration of hospital treatment among RTI patients, associated hospital costs, and focusing on monitoring the long-term consequences of injuries, such as disability, quality of life, and the lasting economic costs for families.

## Conclusion

Based on the results of our research, there is a clear disparity in the incidence rates of road traffic injuries (RTI) across various regions of Serbia, highlighting the need for enhanced traffic safety measures at the local level. Our findings indicate that men are a high-risk group, and there is a significant association between road user categories, age, and gender. These insights can inform the prioritization of targeted preventive measures. Implementing a multisectoral approach—integrating education, law enforcement, infrastructure improvements, and public health campaigns—can reduce the strain on healthcare systems and enhance overall road safety in Serbia.

## Data Availability

The original contributions presented in the study are included in the article/supplementary material, further inquiries can be directed to the corresponding author.
